# CT-based habitat radiomics for preoperative differentiation of adenocarcinoma in situ/minimally invasive adenocarcinoma from invasive adenocarcinoma manifesting as ground-glass nodules: a multicenter study

**DOI:** 10.3389/fonc.2025.1660071

**Published:** 2025-10-15

**Authors:** Ning Dong, Yumao Yan, Yunxin Li, Guochao Li, Ping Wang, Lin Li, Hu Zhang, Hui Sheng, Xiaoyuan Sun

**Affiliations:** ^1^ Department of Radiology, Yantaishan Hospital, Yantai, Shandong, China; ^2^ Department of Radiology, Yantai Qishan Hospital, Yantai, Shandong, China; ^3^ Department of Radiology, Yantai Yuhuangding Hospital, Yantai, Shandong, China; ^4^ Department of Radiology, Affiliated Hospital of Binzhou Medical University, Binzhou, Shandong, China

**Keywords:** computed tomography, habitat, lung adenocarcinoma, radiomics, ground-glass nodules

## Abstract

**Objectives:**

To develop a CT-based habitat radiomics model for preoperative differentiation of adenocarcinoma in situ/minimally invasive adenocarcinoma (AIS/MIA) from invasive adenocarcinoma (IAC) manifesting as ground-glass nodules (GGNs), and to construct a combined model integrating clinical risk factors for optimizing individualized treatment decisions.

**Methods:**

We retrospectively collected imaging and clinical data from 630 patients with pathologically confirmed ground-glass nodules (GGNs) who underwent surgical resection at two medical centers between January 2020 and December 2024. Patients from Center 1 were randomly divided into training and internal validation sets at a 7:3 ratio, while patients from Center 2 served as the external validation set. Tumor habitats were generated using K-means clustering, and radiomics features were extracted from intratumoral, peritumoral 1mm, peritumoral 2mm, and habitat regions. Feature selection was performed using Least Absolute Shrinkage and Selection Operator (LASSO) regression, and predictive models were constructed using multiple machine learning algorithms. A combined nomogram was developed by integrating the Habitat model, Intratumoral model, and Clinic model. Model performance was evaluated using receiver operating characteristic (ROC) curves, calibration curves, and decision curve analysis (DCA).

**Results:**

In the training set, the Combined model demonstrated optimal performance (AUC = 0.928), followed by the Habitat model (AUC = 0.924), both significantly outperforming the Intratumoral model (AUC = 0.879), Peritumoral 1mm model (AUC = 0.874), Peritumoral 2mm model (AUC = 0.868), and Clinic model (AUC = 0.807) (P<0.05). In the external validation set, the Combined model maintained superior performance (AUC = 0.897), significantly exceeding all other models (P<0.05). The Habitat model showed the second-best performance in external validation (AUC = 0.840). Hosmer-Lemeshow test and calibration curves demonstrated good calibration for both the Combined and Habitat models across all cohorts. DCA indicated high net benefit for both models in clinical applications.

**Conclusion:**

CT-based habitat radiomics effectively quantifies intratumoral heterogeneity, significantly improving the differentiation between AIS/MIA and IAC. The combined nomogram integrating habitat features, intratumoral features, and clinical factors demonstrates excellent diagnostic performance and generalizability, providing a reliable preoperative assessment tool for individualized treatment decision-making in ground-glass nodular lung adenocarcinoma.

## 1 Introduction

Lung cancer remains the most prevalent cancer type globally and the leading cause of cancer-related mortality (1). Lung adenocarcinoma represents the most common histological subtype of lung cancer (2). With the widespread implementation of low-dose computed tomography (CT) in lung cancer screening, the detection rate of ground-glass nodules (GGNs) has increased substantially (3), with GGNs being a common manifestation of lung adenocarcinoma (4). The 2021 World Health Organization Classification of Thoracic Tumors categorizes lung adenocarcinoma into precursor glandular lesions (including atypical adenomatous hyperplasia and adenocarcinoma *in situ* [AIS]), minimally invasive adenocarcinoma (MIA), and invasive adenocarcinoma (IAC) (5). AIS/MIA demonstrates excellent prognosis with a 5-year disease-free survival (DFS) rate of 100% after surgery (6), whereas IAC shows poorer outcomes with 5-year DFS rates ranging from 38% to 86% (7, 8). Surgical approaches also differ significantly: lobectomy remains the standard treatment for IAC, while sublobar resection is preferred for AIS/MIA (9). Therefore, accurate preoperative differentiation between AIS/MIA and IAC is crucial for developing individualized treatment strategies and avoiding overtreatment or undertreatment.

Conventional imaging examinations have limitations in differentiating the invasiveness of GGNs. Although nodule size, morphological features, and density correlate with invasiveness, these qualitative or semi-quantitative assessment methods are subjective and demonstrate limited accuracy in distinguishing AIS/MIA from IAC (10, 11). Radiomics, an emerging artificial intelligence-based imaging analysis approach, efficiently extracts high-throughput feature information from massive medical images, encompassing shape, texture, signal intensity, and numerous other aspects. These rich and detailed features have been widely applied in disease diagnosis, prognosis assessment, and treatment response monitoring, demonstrating significant clinical value and development potential (12–14). Recent years have witnessed substantial progress in CT radiomics-based differentiation of AIS/MIA from IAC. These studies provide important evidence for early diagnosis and treatment decision-making of IAC by extracting and analyzing high-dimensional features from CT images (8, 15–17). Despite their innovation and promising predictive performance, these studies treat the entire tumor as a single region of interest (ROI) for feature extraction, overlooking the significant heterogeneity characteristic of ground-glass nodular lung adenocarcinoma (18).

The tumor microenvironment plays a pivotal role in shaping tumor heterogeneity. The diversity of stromal cell types and functional heterogeneity directly sculpts the complex environmental landscape within tumors (19). Stromal components including cancer-associated fibroblasts, tumor-associated macrophages, and vascular endothelial cells create spatially heterogeneous microenvironmental gradients through secretion of different growth factors and cytokines. This spatial microenvironmental heterogeneity further promotes adaptive evolution of tumor cells under selective pressure, leading to the emergence of tumor cell subpopulations with different phenotypic and functional characteristics, ultimately forming complex patterns of intratumoral heterogeneity (20). Given this inherent spatial complexity within tumors, traditional radiomics approaches that analyze tumors as single homogeneous entities may inadequately capture the full spectrum of biological diversity present in these heterogeneous tissues (21, 22). To address this limitation and better reflect the spatial complexity of tumor biology, habitat radiomics quantifies intratumoral heterogeneity by segmenting complex tumors into distinct subregions (called habitats) (23). This approach overcomes the limitation of traditional radiomics that treats tumors as homogeneous entities, enabling deeper analysis of biological differences between tumor regions and providing more reliable imaging evidence for personalized treatment strategies (24). Multiple studies have demonstrated the promising application value of habitat radiomics in predicting glioma molecular markers, Human Epidermal Growth Factor Receptor 2 expression status in breast cancer, and lymphovascular space invasion in cervical cancer (25–27). The peritumoral region, as an integral component of the tumor microenvironment, contains information related to tumor molecular subtypes, invasiveness, and lymph node metastasis, holding significant value in tumor molecular subtyping, prognosis assessment, and metastasis prediction (28–30).

This study aims to develop a CT-based habitat radiomics model for differentiating AIS/MIA from IAC manifesting as GGNs. Furthermore, we integrate the habitat model with intratumoral (or peritumoral) features and clinical risk factors to construct a combined nomogram model, providing clinicians with more comprehensive and accurate diagnostic evidence to optimize individualized treatment decision-making.

## 2 Materials and methods

### 2.1 Patients

**Figure 1:**
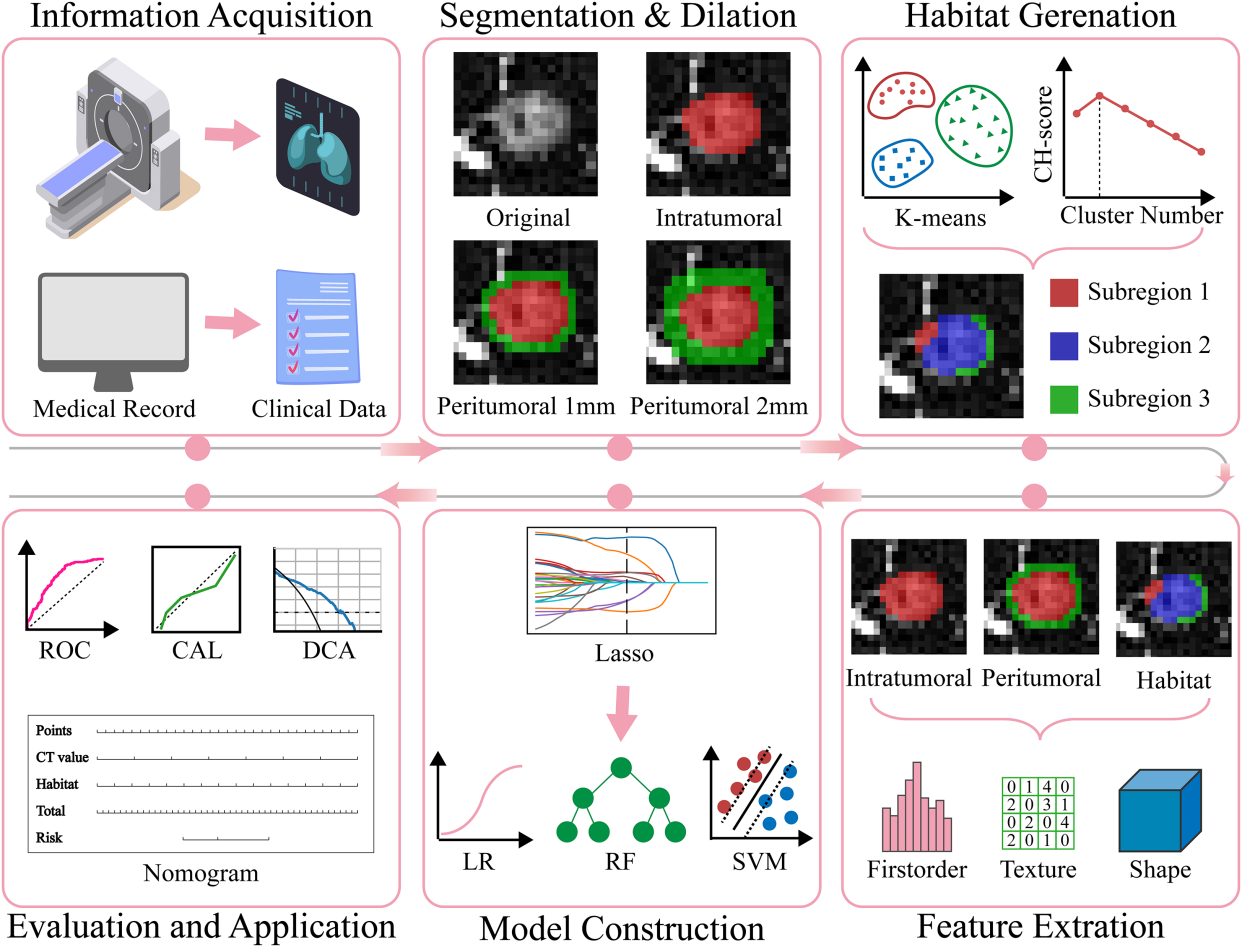
The overall workflow of this study. CAL, calibration; CH, Calinski-Harabasz; DCA, decision curve analysis; Lasso, least absolute shrinkage and selection operator; LR, Logistic Regression; RF, Random Forest; ROC, receiver operating characteristic; SVM, Support Vector Machine.

This multicenter study was approved by the ethics committees of Yantaishan Hospital and Affiliated Hospital of Binzhou Medical University. Given the retrospective nature of this study, the requirement for informed consent was waived. **Figure 1** illustrates the specific workflow of this study.

**Figure 2:**
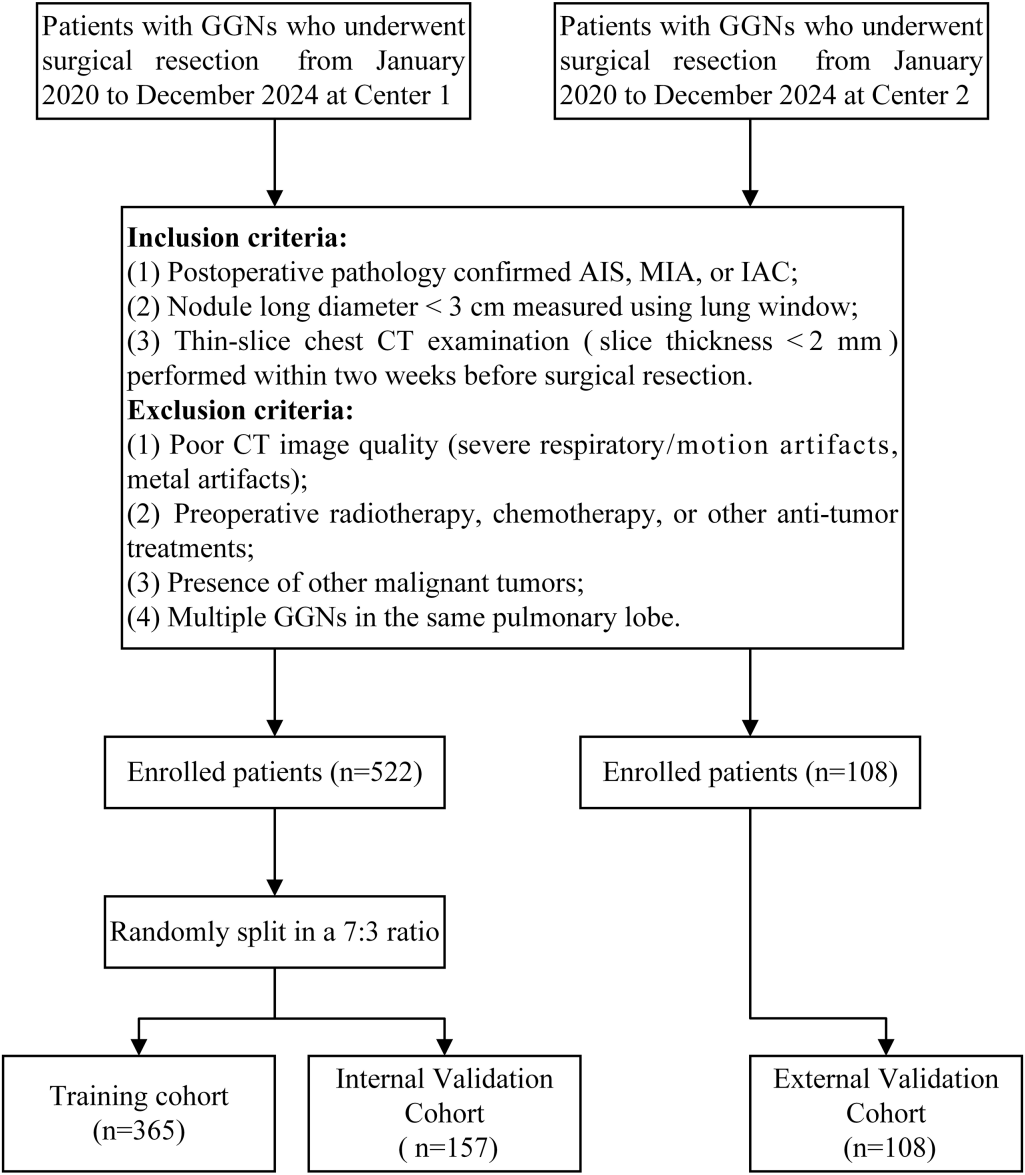
Flowchart of inclusion and exclusion criteria.

We retrospectively collected imaging and clinical data from patients with GGNs who underwent surgical resection at Center 1 (Yantaishan Hospital) and Center 2 (Affiliated Hospital of Binzhou Medical University) between January 2020 and December 2024. Inclusion criteria were as follows (1): pathologically confirmed AIS, MIA, or IAC after surgery (2); nodule long diameter <3 cm measured on lung window (window width: 1200 Hounsfield Units [HU]; window level: -600 HU) (3); thin-slice chest CT examination within two weeks before surgical resection, with slice thickness less than 2 mm. Exclusion criteria were (1): poor CT image quality (severe respiratory artifacts, metal artifacts, etc.) (2); previous radiotherapy, chemotherapy, or other antitumor treatment (3); concomitant other malignancies (4); multiple GGNs in the same lobe. Ultimately, 630 GGNs from 630 eligible patients were included in this study. The 522 GGNs from Center 1 were randomly divided into training and internal validation sets at a 7:3 ratio, while the 108 GGNs from Center 2 served as the external validation set (**Figure 2**).

### 2.2 Image acquisition and preprocessing

This study employed a multicenter imaging acquisition protocol. Both centers were equipped with CT scanners from Philips Medical Systems (Cleveland, USA), including Brilliance 64, Brilliance 128, and Incisive 64. All patients received standardized breathing training before scanning and were positioned supine (head first, arms raised and placed beside the head). Scanning was performed at maximum inspiratory breath-hold. For the pulmonary nodule region, a targeted scanning protocol was used to obtain non-contrast high-resolution images with the following parameters: tube voltage 120 kV, tube current 300 mA, pitch 0.6, collimation 0.625 mm × 64, matrix size 1024 × 1024, field of view 200 mm, reconstruction slice thickness 0.670 mm, reconstruction slice interval 0.340 mm, and sharp reconstruction algorithm. To reduce inter-equipment variability and improve the comparability and reproducibility of radiomics features, thereby enhancing model robustness and generalizability, voxel spacing was first resampled to 1 mm × 1 mm × 1 mm using nearest neighbor interpolation, followed by histogram standardization of intensity values.

### 2.3 ROI segmentation and peritumoral region generation

A junior radiologist A (5 years of experience in chest imaging diagnosis) used ITK-SNAP software (version 3.8.0; http://www.itksnap.org) to manually delineate ROI along nodule edges layer by layer under lung window settings (window width: 1200 HU; window level: -600 HU) until the entire nodule was covered, obtaining three-dimensional volume of interest (VOI). Large vessels and bronchi within nodules were carefully excluded during delineation. Subsequently, a senior radiologist B (20 years of experience in chest imaging diagnosis) reviewed the delineation results. Disagreements between the two radiologists were resolved through consensus. Both radiologists were blinded to pathological results throughout the process to ensure objectivity. Finally, using the VOI outer surface as a reference, morphological dilation algorithms were applied to generate peritumoral regions extending 1 mm and 2 mm outward. Non-lung tissues such as chest wall, ribs, and heart covered during the dilation process were manually excluded.

### 2.4 Habitat generation

**Figure 3:**
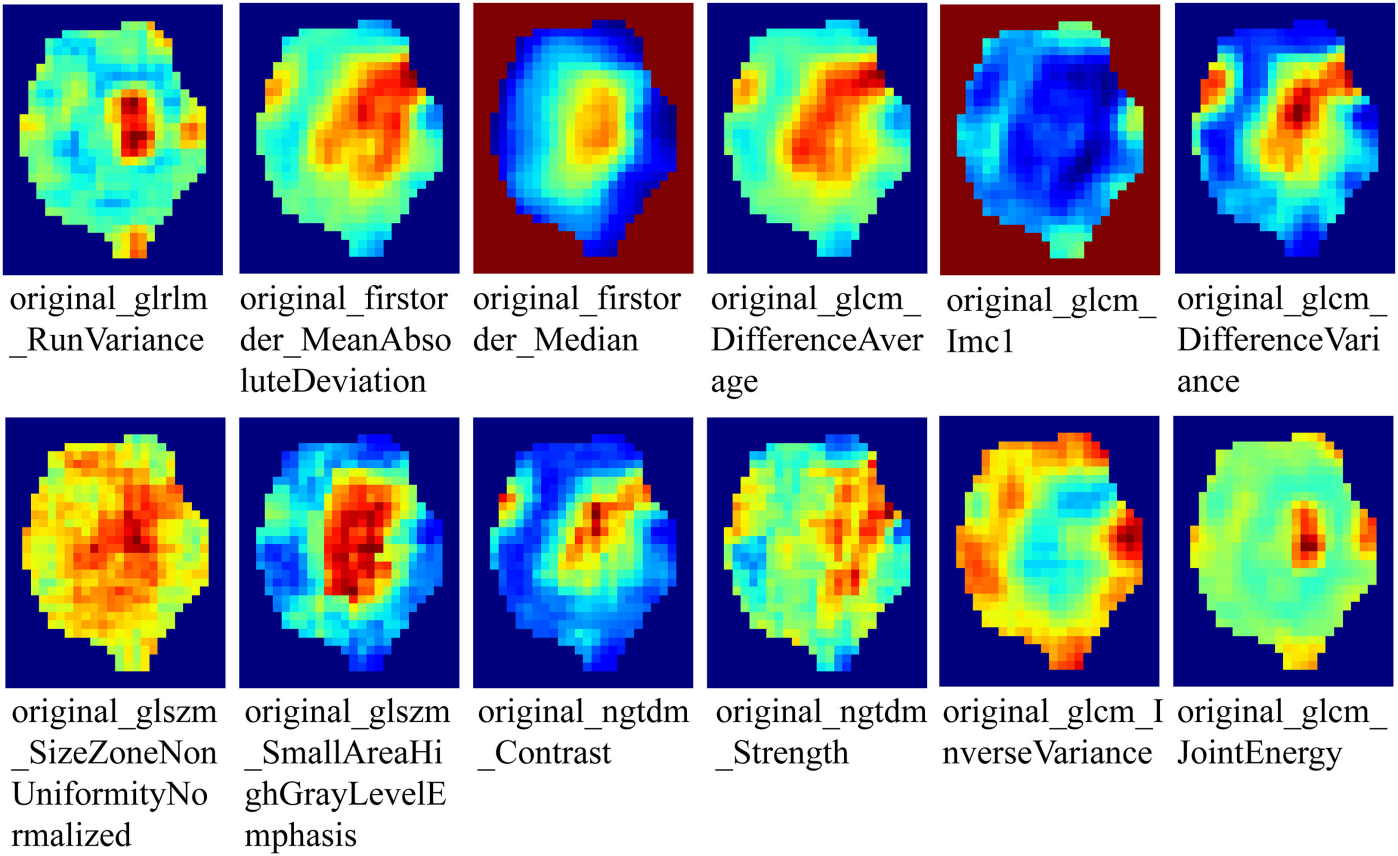
Visualization of local features of voxels within the VOI.

To generate tumor habitats, 12 local features were extracted from each voxel within the three-dimensional VOI (**Figure 3** shows feature visualization), followed by K-means clustering to delineate habitat regions. Cluster numbers from 2 to 9 were evaluated, with the optimal number selected based on Calinski-Harabasz scores (31). Specific details regarding habitat generation are provided in the Supplementary Materials.

### 2.5 Feature extraction and selection

Multi-regional radiomics feature extraction was performed using the PyRadiomics platform (version 3.0.1), including (1): intratumoral region (2); peritumoral 1mm region (3); peritumoral 2mm region (4); tumor habitat regions. Feature extraction strictly followed the Imaging Biomarker Standardization Initiative guidelines (32), encompassing three major categories (1): first-order statistics features, characterizing signal intensity distribution (2); shape features, quantifying spatial geometric attributes of lesions (3); higher-order texture features, analyzing inter-pixel correlation patterns through Gray Level Co-occurrence Matrix, Gray Level Dependence Matrix, Gray Level Run Length Matrix, Gray Level Size Zone Matrix, and Neighboring Gray Tone Difference Matrix to characterize microscopic heterogeneity.

To assess feature extraction consistency and reproducibility, 30 GGNs were randomly selected for independent ROI segmentation by radiologists A and B, with interclass correlation coefficients calculated; 2 weeks later, radiologist A repeated segmentation of the same nodules to calculate intraclass correlation coefficients. Features with both intraclass and interclass correlation coefficients greater than 0.75 were retained for subsequent analysis. Due to the unsupervised nature of clustering, this process was omitted for habitat model feature selection. Feature values were standardized using Z-score normalization based on the mean and standard deviation of the training cohort to eliminate scale effects. Features with p<0.05 by t-test were retained. Pearson correlation coefficients were calculated to identify highly correlated features, with a threshold of 0.9. The minimum Redundancy Maximum Relevance algorithm was used to select the top 30 features most relevant to outcomes with low mutual redundancy. To further improve model generalizability, the Least Absolute Shrinkage and Selection Operator (LASSO) regression model was constructed on the training set, with the optimal regularization parameter λ determined through 10-fold cross-validation. Features with non-zero coefficients based on the optimal λ value were selected for final predictive model construction.

### 2.6 Model construction

In this study, we constructed the following four radiomics models based on different regions (1): Intratumoral (Intra) model (2); Peritumoral 1mm (Peri 1mm) model (3); Peritumoral 2mm (Peri 2mm) model (4); Habitat model. For Clinic model construction, we first performed univariate logistic regression analysis on all clinical and imaging variables, selecting variables with p<0.05, followed by multivariate logistic regression analysis to identify independent risk factors for IAC for modeling. For radiomics and clinic model construction, we employed various advanced machine learning algorithms, including Logistic Regression, Support Vector Machine, Random Forest, eXtreme Gradient Boosting, and Light Gradient Boosting Machine. To ensure model performance and stability, we used five-fold cross-validation and grid search algorithms to determine optimal hyperparameters for each algorithm. To construct the combined model, we performed a comprehensive evaluation of model performance, complementarity, and clinical applicability. The Habitat model demonstrated superior performance with strong generalizability (external validation AUC = 0.840), while the Intra model provided comprehensive tumor characterization (external validation AUC = 0.756). Although the peritumoral models showed predictive ability, their relatively lower performance (Peri 1mm external validation AUC = 0.747; Peri 2mm external validation AUC = 0.730) led to their exclusion from the final combined model. Additionally, including too many radiomics models could increase model complexity and risk of overfitting. Finally, the Intra model, Habitat model, and Clinic model were integrated to construct a combined model, visualized in nomogram form.

### 2.7 Model evaluation

Model performance was evaluated using receiver operating characteristic (ROC) curve metrics, specifically including area under the curve (AUC), accuracy, sensitivity, specificity, positive predictive value, and negative predictive value. Through comparison and analysis of different machine learning algorithms, the algorithm with the maximum AUC in the internal validation set was selected as the basis for constructing corresponding radiomics and Clinic models. To validate differences in predictive performance between models, pairwise comparisons were performed using the DeLong test. For model calibration assessment, calibration curves were used to visually present the consistency between predicted probabilities and actual occurrence probabilities, with the Hosmer-Lemeshow test providing quantitative assessment of calibration ability. Decision curve analysis (DCA) was employed to evaluate the clinical net benefit of models at different risk thresholds.

### 2.8 Statistical analysis

Statistical analysis was performed using SPSS (version 26.0) and Python (version 3.9.7). Continuous variables were expressed as mean ± standard deviation, with between-group comparisons using independent sample t-tests. Categorical variables were expressed as frequencies and percentages, with between-group differences compared using chi-square tests or Fisher’s exact test. All statistical tests were two-sided, with p<0.05 considered statistically significant.

## 3 Results

### 3.1 Patient characteristics

This study included 630 patients from two centers, comprising 365 patients in the training set (mean age 56.46 ± 11.59 years), 157 patients in the internal validation set (mean age 55.79 ± 11.43 years), and 108 patients in the external validation set (mean age 53.75 ± 10.75 years). Age, long diameter, short diameter, CT value, lobulation, spiculation, vessel changes, shape, and type showed statistically significant differences between AIS/MIA and IAC groups across all three cohorts. Specifically, in all cohorts, the IAC group had higher age of onset than the AIS/MIA group, with larger long and short diameters and higher CT values. Additionally, the incidence of lobulation, spiculation, vessel changes, round nodules, and mixed ground-glass nodules was significantly higher in the IAC group than in the AIS/MIA group. Detailed data are presented in **Table 1**.

**Table 1:** Comparison of clinical and imaging characteristics between the AIS/MIA group and the IAC group in the three cohorts.

Features	Training cohort (n=365)		P	Internal validation cohort (n=157)		P	External validation cohort (n=108)		P
	AIS/MIA (n=195)	IAC (n=170)		AIS/MIA (n=85)	IAC (n=72)		AIS/MIA (n=70)	IAC (n=38)	
Age (years)	52.76 ± 12.24	60.69 ± 9.14	<0.001	52.35 ± 11.77	59.85 ± 9.60	<0.001	51.03 ± 10.21	58.76 ± 10.01	<0.001
CEA (ng/ml)	1.74 ± 1.22	2.08 ± 1.17	<0.001	1.83 ± 1.46	3.22 ± 11.17	0.189	1.73 ± 0.81	2.20 ± 1.67	0.256
NSE (ng/ml)	15.53 ± 5.56	15.59 ± 4.58	0.496	14.05 ± 4.15	16.30 ± 6.48	0.040	12.79 ± 3.37	13.05 ± 3.74	0.731
Long diameter (mm)	9.12 ± 2.66	15.64 ± 5.79	<0.001	9.67 ± 3.55	15.20 ± 5.57	<0.001	8.86 ± 4.50	15.40 ± 5.57	<0.001
Short diameter (mm)	7.07 ± 2.07	11.06 ± 4.25	<0.001	7.46 ± 2.74	10.63 ± 3.87	<0.001	7.07 ± 2.15	11.76 ± 4.10	<0.001
CT value (HU)	476.56 ± 175.74	333.11 ± 181.83	<0.001	466.15 ± 180.94	329.00 ± 180.22	<0.001	484.31 ± 184.17	334.37 ± 220.86	<0.001
Gender [n (%)]			0.072			0.457			0.026
Male	71(36.41)	46(27.06)		26(30.59)	27(37.50)		14(20.00)	16(42.11)	
Female	124(63.59)	124(72.94)		59(69.41)	45(62.50)		56(80.00)	22(57.89)	
Smoking [n (%)]			0.770			0.025			0.087
No	175(89.74)	150(88.24)		78(91.76)	56(77.78)		62(88.57)	28(73.68)	
Yes	20(10.26)	20(11.76)		7(8.24)	16(22.22)		8(11.43)	10(26.32)	
Location [n (%)]			0.932			0.436			0.705
RUL	61(31.28)	53(31.18)		29(34.12)	21(29.17)		24(34.29)	14(36.84)	
RML	12(6.15)	12(7.06)		4(4.71)	7(9.72)		4(5.71)	1(2.63)	
RLL	29(14.87)	30(17.65)		16(18.82)	15(20.83)		11(15.71)	9(23.68)	
LUL	58(29.74)	48(28.24)		27(31.76)	17(23.61)		21(30.00)	8(21.05)	
LLL	35(17.95)	27(15.88)		9(10.59)	12(16.67)		10(14.29)	6(15.79)	
Lobulation [n (%)]			<0.001			<0.001			<0.001
No	71(36.41)	11(6.47)		33(38.82)	3(4.17)		36(51.43)	2(5.26)	
Yes	124(63.59)	159(93.53)		52(61.18)	69(95.83)		34(48.57)	36(94.74)	
Spiculation [n (%)]			<0.001			0.003			<0.001
No	156(80.00)	81(47.65)		60(70.59)	33(45.83)		59(84.29)	15(39.47)	
Yes	39(20.00)	89(52.35)		25(29.41)	39(54.17)		11(15.71)	23(60.53)	
Margin [n (%)]			<0.001			0.100			0.003
Clear	140(71.79)	92(54.12)		58(68.24)	39(54.17)		51(72.86)	16(42.11)	
Unclear	55(28.21)	78(45.88)		27(31.76)	33(45.83)		19(27.14)	22(57.89)	
Vessel changes [n (%)]			<0.001			<0.001			<0.001
No	102(52.31)	24(14.12)		44(51.76)	5(6.94)		36(51.43)	5(13.16)	
Yes	93(47.69)	146(85.88)		41(48.24)	67(93.06)		34(48.57)	33(86.84)	
Bubble lucency [n (%)]			0.007			0.057			0.018
No	148(75.90)	106(62.35)		71(83.53)	50(69.44)		49(70.00)	17(44.74)	
Yes	47(24.10)	64(37.65)		14(16.47)	22(30.56)		21(30.00)	21(55.26)	
Pleural retraction [n (%)]			<0.001			<0.001			0.076
No	152(77.95)	65(38.24)		59(69.41)	29(40.28)		55(78.57)	23(60.53)	
Yes	43(22.05)	105(61.76)		26(30.59)	43(59.72)		15(21.43)	15(39.47)	
Shape [n (%)]			<0.001			<0.001			<0.001
Round	106(54.36)	29(17.06)		42(49.41)	11(15.28)		58(82.86)	13(34.21)	
Irregular	89(45.64)	141(82.94)		43(50.59)	61(84.72)		12(17.14)	25(65.79)	
Type [n (%)]			<0.001			<0.001			0.013
pGGN	135(69.23)	48(28.24)		53(62.35)	15(20.83)		16(22.86)	1(2.63)	
mGGN	60(30.77)	122(71.76)		32(37.65)	57(79.17)		54(77.14)	37(97.37)	

AIS, adenocarcinoma *in situ*; CEA, carcinoembryonic antigen; CT, computed tomography; HU, Hounsfield units; IAC, invasive adenocarcinoma; LLL, left lower lobe; LUL, left upper lobe; mGGN, mixed round-glass nodules; MIA, minimally invasive adenocarcinoma; NSE, neuron specific enolase; pGGN, pure ground-glass nodules; RLL, right lower lobe; RML, right middle lobe; RUL, right upper lobe.

### 3.2 Habitat generation

When generating habitat subregions, we evaluated subregion numbers from 2 to 9. As shown in **Supplementary Figure S1**, the Calinski-Harabasz score increased when the number of subregions increased from 2 to 3, then gradually decreased, indicating the optimal number of subregions was 3. The different subregions were named Habitat 1, Habitat 2, and Habitat 3.

### 3.3 Feature selection and model construction

**Figure 4:**
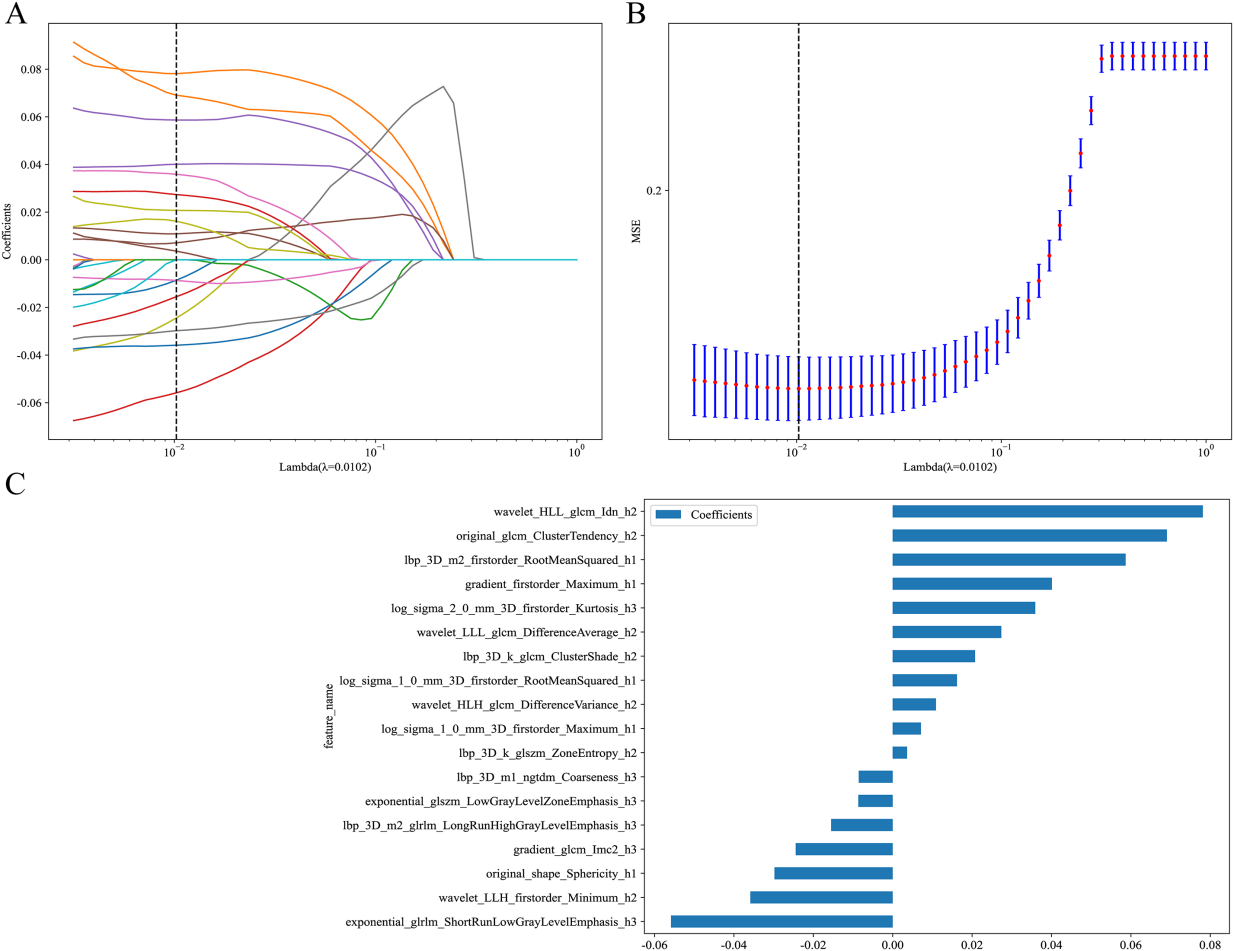
LASSO regression screening of radiomic features in the Habitat model. **(A)** LASSO coefficient path plot. This plot illustrates the trajectories of feature coefficients as the regularization parameter (Lambda) varies. As Lambda increases, the coefficients shrink toward zero, identifying key features at the optimal Lambda value (dashed line). **(B)** LASSO regression MSE curve plot. The dashed line marks the optimal Lambda value where MSE is minimized, determining the final feature subset. **(C)** LASSO-screened feature coefficient distribution plot. This shows the coefficients of features selected by LASSO regression. MSE, mean squared error.

**Figure 5:**
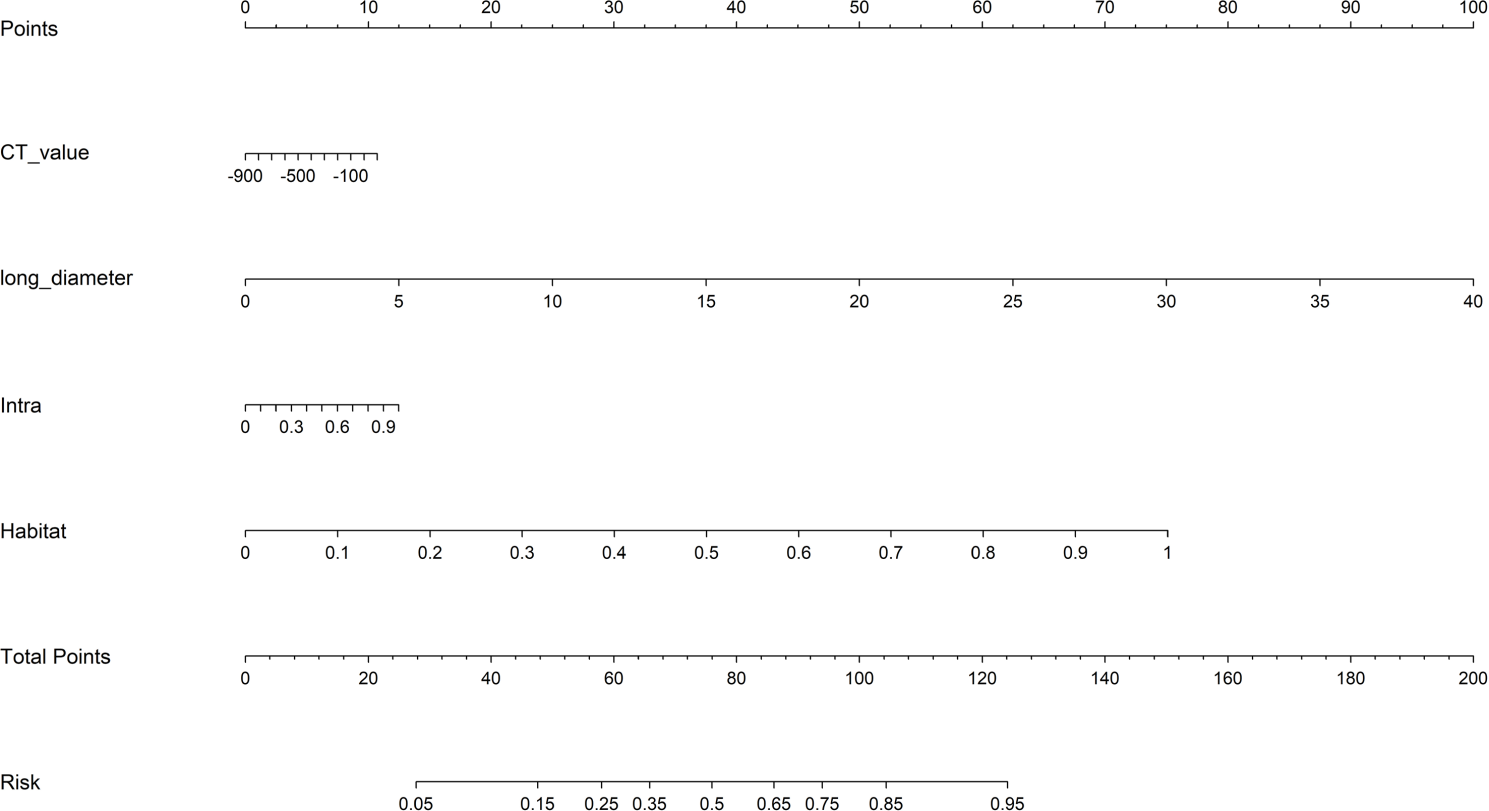
The integrated nomogram model. CT, computed tomography.

From the intratumoral region, peritumoral 1mm region, peritumoral 2mm region, and each habitat subregion, 1834 features were extracted respectively, including first-order features, shape features, and texture features, resulting in a total of 5502 features extracted from the overall tumor habitat regions. Through LASSO screening, 15 features were retained from the intratumoral region, 11 features from the peritumoral 1mm region, 16 features from the peritumoral 2mm region, and 18 features from the habitat regions for corresponding model construction (**Supplementary Figures S2**-**S4**, **Figure 4**). In Clinic model construction, univariate logistic regression showed long diameter, short diameter, CT value, lobulation, spiculation, margin, vessel changes, pleural retraction, shape, and type as potential risk factors. Further multivariate logistic regression identified long diameter and CT value for subsequent modeling (**Table 2**). Based on AUC evaluation results in the internal validation set, the Intra model, Peri 1mm model, and Habitat model all adopted the Logistic Regression algorithm, the Peri 2mm model adopted the eXtreme Gradient Boosting algorithm, and the Clinic model adopted the Random Forest algorithm. Specific performance of each model under different algorithms is detailed in **Supplementary Tables S1**-**S5** and **Supplementary Figure S5**. Finally, the Intra model, Habitat model, and Clinic model were integrated to construct the combined nomogram model (**Figure 5**).

**Table 2:** Univariate and multivariate logistic regression analysis of clinical and imaging variables.

Features	Univariate logistic regression	P	Multivariate logistic regression	P
	OR (95% CI)		OR (95% CI)	
Age	1.000 (0.997-1.003)	0.979		
CEA	1.015 (0.940-1.096)	0.747		
NSE	0.992 (0.982-1.003)	0.228		
Long diameter	1.028 (1.014-1.042)	0.001	1.237 (1.116-1.372)	0.001
Short diameter	1.030(1.011-1.049)	0.007	1.063 (0.932-1.213)	0.442
CT value	1.001 (1.001-1.001)	0	1.007 (1.006-1.009)	0
Gender	1.000 (0.811-1.232)	1.000		
Smoking	1.000 (0.595-1.682)	1.000		
Location	0.947 (0.882-1.016)	0.202		
Lobulation	1.282 (1.053-1.562)	0.038	0.785 (0.409-1.504)	0.540
Spiculation	2.282 (1.664-3.130)	0	1.104 (0.650-1.878)	0.759
Margin	1.418 (1.062-1.895)	0.047	0.760 (0.448-1.289)	0.392
Vessel changes	1.570 (1.262-1.952)	0.001	0.732 (0.433-1.239)	0.330
Bubble lucency	1.362 (0.993-1.868)	0.108		
Pleural retraction	2.442 (1.813-3.290)	0	1.671 (0.999-2.795)	0.101
Shape	1.584 (1.267-1.980)	0.001	1.737 (0.962-3.139)	0.124
Type	2.033 (1.568-2.635)	0	0.700 (0.418-1.172)	0.256

CEA, carcinoembryonic antigen; CI, confidence interval; CT, computed tomography; NSE, neuron specific enolase; OR, odds ratio.

### 3.4 Model performance and evaluation

**Figure 6:**
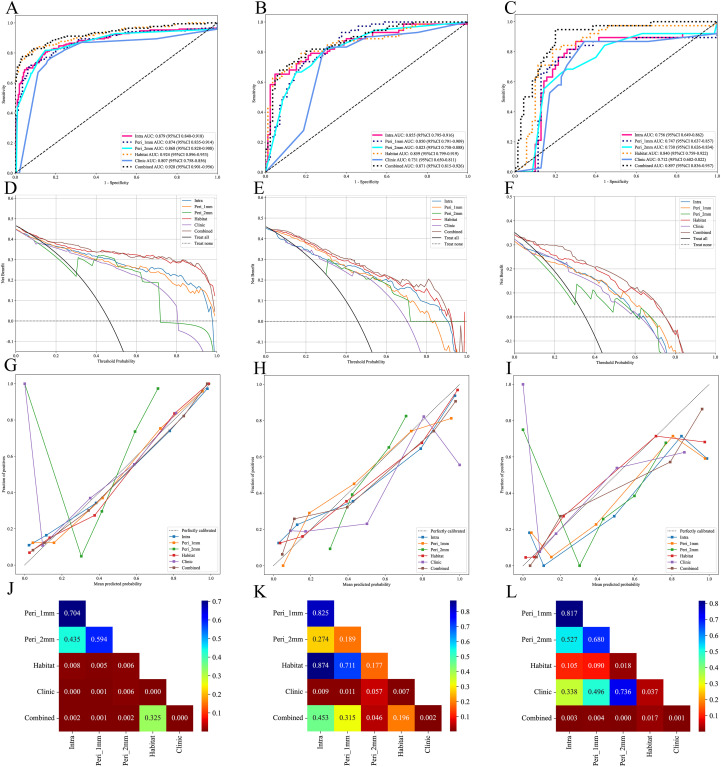
Performance evaluation of different models in the training, internal validation, and external validation cohorts. **(A-C)**, ROC curves of different models in the training, internal validation and external validation cohorts; **(D-F)**, DCA curves of different models in the training, internal validation and external validation cohorts; **(G-I)**, calibration curves of different models in the training, internal validation and external validation cohorts; **(J-L)**, Delong test of different models in the training, internal validation and external validation cohorts.

ROC curve analysis showed that in the training cohort, the combined model exhibited optimal diagnostic performance with an AUC of 0.928 (95% CI: 0.901-0.956) and accuracy of 0.874, ranking first among all models in both AUC and accuracy. The habitat model closely followed with an AUC of 0.924 (95% CI: 0.896-0.953) and accuracy of 0.871. The Intra model, Peri 1mm model, and Peri 2mm model showed similar diagnostic performance (AUC range: 0.868-0.879), all significantly lower than the Combined and Habitat models. The Clinic model achieved only an AUC of 0.807, indicating limited clinical diagnostic value. DeLong test further confirmed that the Combined and Habitat models significantly outperformed the Intra model, Peri 1mm model, Perit 2mm model, and Clinic model (p<0.05). In the internal validation cohort, models performance generally decreased, but the Combined model (AUC: 0.871, 95% CI: 0.815-0.926) and Habitat model (AUC: 0.859, 95% CI: 0.799-0.919) continued to maintain leading advantages. In the external validation cohort, the Combined model demonstrated the most excellent generalization ability, with its AUC (0.897, 95% CI: 0.836-0.957) significantly superior to all other single models (Intra AUC: 0.756, Peri 1mm AUC: 0.747, Peri 2mm AUC: 0.730, Clinic AUC: 0.712, Habitat AUC: 0.840), with DeLong test showing p-values all less than 0.05. Meanwhile, the Habitat model also showed good robustness, being the second-best performing model in external validation. Details are shown in **Table 3** and **Figure 6**.

**Table 3:** Comparison of prediction performance of different models.

Model	AUC (95% CI)	Accuracy	Sensitivity	Specificity	PPV	NPV
Training cohort						
Intra	0.879 (0.840 - 0.918)	0.827	0.800	0.851	0.824	0.830
Peri 1mm	0.874 (0.835 - 0.914)	0.827	0.712	0.928	0.896	0.787
Peri 2mm	0.868 (0.828 - 0.908)	0.822	0.741	0.892	0.857	0.798
Clinic	0.807 (0.758 - 0.856)	0.797	0.747	0.841	0.804	0.792
Habitat	0.924 (0.896 - 0.953)	0.871	0.794	0.938	0.918	0.839
Combined	0.928 (0.901 - 0.956)	0.874	0.771	0.964	0.949	0.828
Internal validation cohort						
Intra	0.855 (0.795 - 0.916)	0.809	0.639	0.953	0.920	0.757
Peri 1mm	0.850 (0.791 - 0.909)	0.758	0.917	0.624	0.673	0.898
Peri 2mm	0.823 (0.758 - 0.888)	0.752	0.764	0.741	0.714	0.787
Clinic	0.731 (0.650 - 0.812)	0.752	0.792	0.718	0.704	0.803
Habitat	0.859 (0.799 - 0.919)	0.803	0.778	0.824	0.789	0.814
Combined	0.871 (0.815 - 0.926)	0.809	0.667	0.929	0.889	0.767
External validation cohort						
Intra	0.756 (0.649 - 0.862)	0.750	0.842	0.700	0.604	0.891
Peri 1mm	0.747 (0.637 - 0.857)	0.769	0.737	0.786	0.651	0.846
Peri 2mm	0.730 (0.626 - 0.834)	0.750	0.553	0.857	0.677	0.779
Clinic	0.712 (0.602 - 0.822)	0.731	0.711	0.743	0.600	0.825
Habitat	0.840 (0.759 - 0.922)	0.806	0.789	0.814	0.698	0.877
Combined	0.897 (0.836 - 0.957)	0.843	0.921	0.800	0.714	0.949

AUC, area under the curve; CI, confidence interval; NPV, negative predictive value; PPV, positive predictive value.

DCA indicated that both the Combined and Habitat models demonstrated high net benefit across all three cohorts, particularly at lower threshold probabilities, suggesting their potential value in early diagnosis. In contrast, while other models showed some clinical benefit within specific threshold probability ranges, their overall performance was notably inferior to the Combined and Habitat models (**Figure 6**).

To evaluate model calibration performance, this study employed Hosmer-Lemeshow test and calibration curves for analysis. Hosmer-Lemeshow test results indicated that only the Combined and Habitat models consistently maintained good calibration ability across training, internal validation, and external validation sets (p>0.05) (**Table 4**). Calibration curves further visualized the predictive accuracy of each model. In the calibration curves, both Habitat and Combined model curves remained close to the ideal calibration line (dashed line) across all three cohorts, indicating high concordance between predicted probabilities and actual observations (**Figure 6**).

**Table 4:** Hosmer-Lemeshow test results for different models in three cohorts.

Cohort	Intra	Peri 1mm	Peri 2mm	Clinic	Habitat	Combined
Training	0.080	1.977×10^-2^	5.737×10^-9^	4.682×10^-7^	0.073	0.350
Internal Validation	0.309	1.272×10^-1^	1.282×10^-1^	3.421×10^-1^	0.063	0.176
External Validation	0.022	1.869×10^-4^	1.274×10^-4^	2.775×10^-4^	0.292	0.131

## 4 Discussion

This study developed and validated a CT-based habitat radiomics model for differentiating AIS/MIA from IAC manifesting as GGNs. The results demonstrate that the habitat radiomics model possesses unique advantages in capturing intratumoral heterogeneity, with diagnostic performance significantly superior to traditional intratumoral and peritumoral radiomics models. The combined nomogram model integrating habitat features, intratumoral features, and clinical risk factors exhibited optimal diagnostic performance, achieving an AUC of 0.897 in the external validation set, providing a reliable quantitative tool for clinical precision diagnosis and treatment.

Accurate preoperative pathological grading of lung adenocarcinoma is crucial for developing individualized treatment strategies. Previous studies have shown that AIS/MIA patients can achieve a 5-year DFS rate of 100% after surgery, while IAC patients have significantly poorer prognosis (7, 33). Therefore, accurate preoperative differentiation between these two lesion types is essential for avoiding overtreatment or undertreatment. This study found that CT value and long diameter were independent risk factors for IAC, consistent with previous research (4, 34). Lung adenocarcinoma generally progresses through four stages: AAH, AIS, MIA, and IAC. During this gradual evolution, increased nodule size often reflects enhanced tumor cell proliferative activity and invasiveness (18). Larger nodules are more likely to contain solid components, typically indicating invasive growth patterns (35). During invasive adenocarcinoma development, tumor cells grow along alveolar walls, initially maintaining alveolar structural integrity with only slight density increases (36). As invasion deepens, tumor cell density increases, fibrous tissue proliferates, and angiogenesis increases, leading to further CT value elevation (37). However, models based solely on clinical factors demonstrated insufficient diagnostic performance (external test set AUC: 0.712), indicating that traditional imaging features alone cannot meet the needs for clinical precision diagnosis.

In recent years, some studies have explored the application value of radiomics in differentiating lung adenocarcinoma pathological subtypes. Zheng et al. (38) constructed a model based on 11 radiomics features achieving an AUC of 0.820 in the training set. Meng et al. (4) selected 8 key features through LASSO regression to establish a Rad-score for differentiating AIS/MIA from IAC, achieving a training set AUC of 0.892. Our Intra model achieved a training set AUC of 0.879, comparable to previous studies but still inferior to the Habitat model’s predictive performance. This study systematically evaluated the diagnostic performance of Peri 1mm and Peri 2mm models (training set AUCs of 0.874 and 0.868, respectively), which, while superior to the Clinic model, were significantly inferior to the Habitat model. These results indicate that although the peritumoral region contains important information related to tumor invasiveness, both intratumoral and peritumoral models analyze these regions as wholes, failing to fully explore their internal heterogeneity information. Additionally, we found that Peri models performed worse than the Intra model, differing from some previous studies (39, 40). Possible reasons for this discrepancy include: first, this study included only GGNs, whose peritumoral microenvironmental changes may be less pronounced than solid nodules; second, the relatively small peritumoral extension distances (1-2mm) selected in this study may not have adequately captured key biological information in the peritumoral region, suggesting that future research should explore larger peritumoral ranges (such as 3-5mm or even broader regions) to comprehensively assess the tumor microenvironment and potentially identify more valuable predictive features.

Traditional radiomics treats tumors as single homogeneous entities for feature extraction, ignoring intratumoral heterogeneity. However, increasing evidence indicates that tumors are highly heterogeneous ecosystems containing cell subpopulations with different phenotypic and functional characteristics (41). Spatial variations in the tumor microenvironment, including oxygen concentration gradients, nutrient distribution, and interstitial pressure differences, drive adaptive evolution of tumor cells, leading to coexistence of cell subpopulations with different proliferative capacities, invasiveness, and treatment sensitivities (19). This heterogeneity is particularly evident in ground-glass nodular lung adenocarcinoma: the tumor center may have already undergone invasion while peripheral regions maintain *in situ* or minimally invasive growth characteristics (42). This study employed K-means clustering based on 12 local features to divide GGNs into 3 habitat subregions, thereby more precisely capturing spatial heterogeneity information within tumors. Results showed that the Habitat model’s predictive performance significantly exceeded other single models (internal validation set AUC: 0.924 *vs* 0.879, 0.874, 0.868, 0.807, all p<0.05), consistent with previous research conclusions. Wu et al. (43) reported that the Habitat model improved AUC by 6.5% compared to traditional radiomics models when predicting epidermal growth factor receptor mutation status in stage I non-small cell lung cancer. Bi et al. (44) reported similar results in predicting drug resistance in ovarian cancer patients. Among the 18 features selected through LASSO for the Habitat model, the top two features—wavelet_HLL_glcm_Idn_h2 and original_glcm_ClusterTendency_h2—are both texture features derived from gray-level co-occurrence matrix (GLCM) analysis. The Inverse Difference Normalized (Idn) feature quantifies local homogeneity in image texture, with higher values indicating more homogeneous regions, which may reflect areas of uniform cellular density characteristic of less invasive tumor components (45). Cluster Tendency measures the grouping of pixels with similar gray-level values, capturing the spatial organization patterns that distinguish between the lepidic growth pattern of AIS/MIA and the more disorganized invasive growth pattern of IAC (46). The prominence of these texture features in our model aligns with pathological observations that IAC exhibits greater architectural complexity and cellular heterogeneity compared to AIS/MIA, manifesting as more heterogeneous texture patterns on CT imaging (47). Notably, the Habitat model demonstrated stable diagnostic performance across all cohorts, particularly in external validation, where performance decline (training set AUC 0.924 *vs* external validation set AUC 0.840) was smaller than other single models. This suggests that habitat features possess stronger generalizability and robustness. The reasons may include: first, habitat features reflect tumor microenvironmental heterogeneity, capturing complex spatial distribution patterns within tumors, with this heterogeneity information remaining relatively stable across different patient populations (48); second, habitat analysis segments tumors into subregions with similar phenotypic characteristics through clustering methods, and this quantification of spatial heterogeneity more accurately reflects tumor biological properties, providing better transferability between different centers (49).

The combined nomogram model integrating habitat features, intratumoral features, and clinical risk factors demonstrated optimal diagnostic performance across all cohorts. The advantages of this multi-dimensional information fusion strategy include: first, different types of features provide complementary diagnostic information, with habitat features capturing intratumoral spatial heterogeneity, intratumoral features reflecting overall attributes, and clinical features providing macroscopic morphological information (50); second, the nomogram format is intuitive and user-friendly, enabling clinicians to quickly assess individual patient IAC risk probability and provide quantitative evidence for clinical decision-making; third, the model maintained good performance in external validation (AUC = 0.897), demonstrating feasibility for cross-center application. DCA showed that the Combined model generated clinical net benefit across a wide range of threshold probabilities, particularly excelling in low threshold intervals. This holds significant importance for GGN management, as early identification of IAC can guide timely surgical intervention and prevent disease progression. Simultaneously, accurate identification of AIS/MIA can avoid overtreatment, reduce unnecessary lobectomies, and preserve more lung function.

This study has certain limitations. First, selection bias inherent to retrospective studies may affect result reliability. To further validate the clinical application value of the constructed models, future large-sample, multicenter prospective studies are necessary, employing rigorous study design and standardized data collection processes to improve evidence level and clinical credibility. Second, the sample size used for inter- and intra-observer consistency assessment (n=30) was relatively limited, which may not fully capture the variability in feature extraction reproducibility across a broader range of cases. Future studies should employ larger sample sizes for consistency evaluation to enhance the robustness of reproducibility assessment. Third, this study analyzed only non-contrast CT images, failing to fully utilize rich information provided by multimodal functional imaging such as contrast-enhanced CT and Positron Emission Tomography-CT. These imaging techniques provide important information about tissue perfusion and metabolic activity, and integrating multimodal imaging data could significantly improve model diagnostic accuracy and clinical utility (51). Last, current habitat generation relies primarily on unsupervised clustering algorithms. While capable of identifying subregions with different imaging characteristics, it lacks direct validation against pathological gold standards. Although these habitat subregions theoretically may reflect different microenvironments within tumors, the accuracy of these correspondences requires validation through systematic imaging-pathology correlation studies to provide a more solid biological theoretical foundation for clinical translation of habitat radiomics technology.

This study successfully constructed a CT-based habitat radiomics diagnostic model achieving precise assessment of ground-glass nodular lung adenocarcinoma invasiveness. Habitat analysis significantly improved diagnostic accuracy by capturing intratumoral spatial heterogeneity information. The nomogram model combining clinical risk factors demonstrated excellent performance and clinical applicability in multicenter validation. This innovative approach provides a new tool for early precision diagnosis of lung adenocarcinoma, with potential to improve patient treatment decisions and clinical outcomes.

## Data Availability

The raw data supporting the conclusions of this article will be made available by the authors, without undue reservation.
