# Supplementary material for: CT-based habitat radiomics for preoperative differentiation of adenocarcinoma in situ/minimally invasive adenocarcinoma from invasive adenocarcinoma manifesting as ground-glass nodules: a multicenter study
Source: Front Oncol. 2025 Oct 15;15:1660071. doi: 10.3389/fonc.2025.1660071 (PMC12568381; doi:10.3389/fonc.2025.1660071)
Supplement: Supplementary file 1 [file DataSheet1.docx]

Supplementary Material

**1 Methods: Habitat generation**

**1.1 Local features extraction**

We employ a 3×3×3 moving window to extract 12 local features from each voxel within the three-dimensional volume of interest (VOI). This approach aims to comprehensively capture multidimensional information characteristics of voxels, providing a rich data foundation for subsequent analysis and enabling precise subregion delineation. These local features include:

- firstorder_Mean Absolute Deviation (MAD)

$$MAD=\frac{1}{N_{p}}\sum_{i=1}^{N_{p}} \left| X\left( i \right)-\bar{X} \right|$$

Mean Absolute Deviation is the mean distance of all intensity values from the Mean Value of the image array.

- firstorder_ Median

The median gray level intensity within the ROI.

- glcm_Difference Average

$$Difference Average=\sum_{k=0}^{N_{g}} kp_{x-y}(k)$$

Difference Average measures the relationship between occurrences of pairs with similar intensity values and occurrences of pairs with differing intensity values.

- glcm_Difference Variance

$$Difference Variance=\sum_{k=0}^{N_{g}-1} \left( k-DA \right)^{2}p_{x-y}(k)$$

Difference Variance is a measure of heterogeneity that places higher weights on differing intensity level pairs that deviate more from the mean.

- glcm_Inverse Variance

$$Inverse Variance=\sum_{k=1}^{N_{g}-1} \frac{p_{x-y}(k)}{k^{2}}$$

Note that k=0 is skipped, as this would result in a division by 0.

- glcm_Joint Energy
- $Joint Energy=\sum_{i=1}^{N_{g}} \sum_{j=1}^{N_{g}} {(p(i,j))}^{2}$

Energy is a measure of homogeneous patterns in the image. A greater Energy implies that there are more instances of intensity value pairs in the image that neighbor each other at higher frequencies.

- glrlm_Run Variance (RV)

$$RV=\sum_{i=1}^{N_{g}} \sum_{j=1}^{N_{r}} {p(i,\left. j \right|\theta)(j-\mu)}^{2}$$

$$\mu=\sum_{i=1}^{N_{g}} \sum_{j=1}^{N_{r}} p(i,\left. j \right|\theta)j$$

RV is a measure of the variance in runs for the run lengths.

- glszm_Size-Zone Non-Uniformity Normalized (SZNN)
- $SZNN=\frac{\sum_{j=1}^{N_{s}} {(\sum_{i=1}^{N_{g}} P(i,j))}^{2}}{N_{z}^{2}}$

SZNN measures the variability of size zone volumes throughout the image, with a lower value indicating more homogeneity among zone size volumes in the image. This is the normalized version of the SZN formula.

- glszm_ Small Area High Gray Level Emphasis (SAHGLE)

$$SAHGLE=\frac{\sum_{i=1}^{N_{g}} \sum_{j=1}^{N_{s}} \frac{P(i,j)i^{2}}{j^{2}}}{N_{z}}$$

SAHGLE measures the proportion in the image of the joint distribution of smaller size zones with higher gray-level values.

Other features such as glcm_Imc1, ngtdm_Contrast, ngtdm_Strength can be found at https://pyradiomics.readthedocs.io/。

**1.2 K-means subregion clustering**

K-means is a distance-based clustering algorithm that aims to partition n objects into k clusters in such a way that the objects within each cluster are as closely related as possible. For each voxel within the VOI, the Euclidean distance between its 12-dimensional feature vector and each cluster centroid is calculated. The voxel is then assigned to the cluster whose centroid is closest. Through iterative updating of the cluster centroids, k subregions within the VOI are ultimately generated.

The objective function of the K-means algorithm is defined as follows:

$$J=\sum_{i=1}^{K} \sum_{j=1}^{n} \left\| x_{j}-\mu_{i} \right\|^{2}$$

Where:

- *J* is the sum of squared errors within clusters.
- *K* is the number of clusters.
- *n* is the total number of data points.
- $x_{j}$ is the j-th point in the dataset.
- $\mu_{i}$ is the center of the i-th cluster.

The voxels within each subregion exhibit similarity in the 12 local features, thereby providing a feature-similarity-based subregion segmentation result for subsequent habitat analysis. The clustering numbers ranging from 2 to 9 were evaluated, and the optimal clustering number was selected based on the Calinski-Harabasz score.

# 2 Supplementary Tables

Table S1. Performance of different machine learning algorithms in Intra model

| Intra Model | AUC (95% CI) | Accuracy | Sensitivity | Specificity | PPV | NPV |
| --- | --- | --- | --- | --- | --- | --- |
| Training Cohort | | | | | | |
| LR | 0.879 (0.840 - 0.918) | 0.827 | 0.800 | 0.851 | 0.824 | 0.830 |
| SVM | 0.745 (0.696 - 0.795) | 0.696 | 0.788 | 0.615 | 0.641 | 0.769 |
| RF | 0.848 (0.805 - 0.891) | 0.814 | 0.712 | 0.903 | 0.864 | 0.782 |
| XGBoost | 0.858 (0.818 - 0.898) | 0.792 | 0.576 | 0.979 | 0.961 | 0.726 |
| LightGBM | 0.868 (0.831 - 0.904) | 0.811 | 0.688 | 0.918 | 0.880 | 0.772 |
| Internal Validation Cohort | | | | | | |
| LR | 0.855 (0.795 - 0.916) | 0.809 | 0.639 | 0.953 | 0.920 | 0.757 |
| SVM | 0.751 (0.674 - 0.829) | 0.707 | 0.694 | 0.718 | 0.676 | 0.735 |
| RF | 0.811 (0.743 - 0.880) | 0.764 | 0.611 | 0.894 | 0.830 | 0.731 |
| XGBoost | 0.817 (0.752 - 0.883) | 0.758 | 0.542 | 0.941 | 0.886 | 0.708 |
| LightGBM | 0.810 (0.744 - 0.876) | 0.752 | 0.528 | 0.941 | 0.884 | 0.702 |
| External Validation Cohort | | | | | | |
| LR | 0.756 (0.649 - 0.862) | 0.750 | 0.842 | 0.700 | 0.604 | 0.891 |
| SVM | 0.793 (0.706 - 0.880) | 0.741 | 0.684 | 0.771 | 0.619 | 0.818 |
| RForest | 0.729 (0.617 - 0.841) | 0.750 | 0.763 | 0.743 | 0.617 | 0.852 |
| XGBoost | 0.761 (0.655 - 0.866) | 0.787 | 0.632 | 0.871 | 0.727 | 0.813 |
| LightGBM | 0.712 (0.601 - 0.824) | 0.759 | 0.711 | 0.786 | 0.643 | 0.833 |

AUC, area under the curve; CI, confidence interval; LightGBM, Light Gradient Boosting Machine; LR, Logistic Regression; NPV, negative predictive value; PPV, positive predictive value; RF, Random Forest; SVM, Support Vector Machine; XGBoost, eXtreme Gradient Boosting.

Table S2. Performance of different machine learning algorithms in Peri 1mm model

| Peri 1mm Model | AUC (95% CI) | Accuracy | Sensitivity | Specificity | PPV | NPV |
| --- | --- | --- | --- | --- | --- | --- |
| Training Cohort | | | | | | |
| LR | 0.874 (0.8354- 0.914) | 0.827 | 0.712 | 0.928 | 0.896 | 0.787 |
| SVM | 0.850 (0.807 - 0.893) | 0.822 | 0.812 | 0.831 | 0.807 | 0.835 |
| RandomForest | 0.861 (0.821 - 0.901) | 0.795 | 0.759 | 0.826 | 0.791 | 0.797 |
| XGBoost | 0.871 (0.835 - 0.907) | 0.814 | 0.694 | 0.918 | 0.881 | 0.775 |
| LightGBM | 0.856 (0.814 - 0.898) | 0.819 | 0.735 | 0.892 | 0.856 | 0.795 |
| Internal Validation Cohort | | | | | | |
| LR | 0.850 (0.791 - 0.909) | 0.758 | 0.917 | 0.624 | 0.673 | 0.898 |
| SVM | 0.835 (0.771 - 0.898) | 0.771 | 0.806 | 0.741 | 0.725 | 0.818 |
| RandomForest | 0.784 (0.711 - 0.857) | 0.745 | 0.736 | 0.753 | 0.716 | 0.771 |
| XGBoost | 0.787 (0.719 - 0.856) | 0.739 | 0.681 | 0.788 | 0.731 | 0.744 |
| LightGBM | 0.804 (0.735 - 0.873) | 0.752 | 0.694 | 0.800 | 0.746 | 0.756 |
| External Validation Cohort | | | | | | |
| LR | 0.747 (0.637 - 0.857) | 0.769 | 0.737 | 0.786 | 0.651 | 0.846 |
| SVM | 0.744 (0.636 - 0.852) | 0.704 | 0.816 | 0.643 | 0.554 | 0.865 |
| RandomForest | 0.793 (0.695 - 0.891) | 0.815 | 0.763 | 0.843 | 0.725 | 0.868 |
| XGBoost | 0.699 (0.594 - 0.805) | 0.731 | 0.711 | 0.743 | 0.600 | 0.825 |
| LightGBM | 0.738 (0.632 - 0.844) | 0.722 | 0.684 | 0.743 | 0.591 | 0.812 |

AUC, area under the curve; CI, confidence interval; LightGBM, Light Gradient Boosting Machine; LR, Logistic Regression; NPV, negative predictive value; PPV, positive predictive value; RF, Random Forest; SVM, Support Vector Machine; XGBoost, eXtreme Gradient Boosting.

Table S3. Performance of different machine learning algorithms in Peri 2mm model

| Peri 2mm Model | AUC (95% CI) | Accuracy | Sensitivity | Specificity | PPV | NPV |
| --- | --- | --- | --- | --- | --- | --- |
| Training Cohort | | | | | | |
| LR | 0.859 (0.816 - 0.902) | 0.833 | 0.741 | 0.913 | 0.881 | 0.802 |
| SVM | 0.837 (0.795 - 0.879) | 0.778 | 0.771 | 0.785 | 0.757 | 0.797 |
| RandomForest | 0.844 (0.801 - 0.887) | 0.756 | 0.506 | 0.974 | 0.945 | 0.693 |
| XGBoost | 0.868 (0.828 - 0.908) | 0.822 | 0.741 | 0.892 | 0.857 | 0.798 |
| LightGBM | 0.843 (0.799 - 0.887) | 0.795 | 0.735 | 0.846 | 0.806 | 0.786 |
| Internal Validation Cohort | | | | | | |
| LR | 0.820 (0.754 - 0.886) | 0.764 | 0.847 | 0.694 | 0.701 | 0.843 |
| SVM | 0.729 (0.650- 0.807) | 0.675 | 0.681 | 0.671 | 0.636 | 0.712 |
| RandomForest | 0.770 (0.697 - 0.843) | 0.707 | 0.625 | 0.776 | 0.703 | 0.710 |
| XGBoost | 0.823 (0.758 - 0.888) | 0.752 | 0.764 | 0.741 | 0.714 | 0.787 |
| LightGBM | 0.820 (0.755 - 0.885) | 0.764 | 0.667 | 0.847 | 0.787 | 0.750 |
| External Validation Cohort | | | | | | |
| LR | 0.765 (0.662 - 0.868) | 0.778 | 0.789 | 0.771 | 0.652 | 0.871 |
| SVM | 0.729 (0.630 - 0.829) | 0.704 | 0.658 | 0.729 | 0.568 | 0.797 |
| RandomForest | 0.746 (0.640 - 0.852) | 0.769 | 0.605 | 0.857 | 0.697 | 0.800 |
| XGBoost | 0.730 (0.626 - 0.834) | 0.750 | 0.553 | 0.857 | 0.677 | 0.779 |
| LightGBM | 0.735 (0.631 - 0.840) | 0.750 | 0.605 | 0.829 | 0.657 | 0.795 |

AUC, area under the curve; CI, confidence interval; LightGBM, Light Gradient Boosting Machine; LR, Logistic Regression; NPV, negative predictive value; PPV, positive predictive value; RF, Random Forest; SVM, Support Vector Machine; XGBoost, eXtreme Gradient Boosting.

Table S4. Performance of different machine learning algorithms in Habitat model

| Habitat Model | AUC (95% CI) | Accuracy | Sensitivity | Specificity | PPV | NPV |
| --- | --- | --- | --- | --- | --- | --- |
| Training Cohort | | | | | | |
| LR | 0.924 (0.896 - 0.953) | 0.871 | 0.794 | 0.938 | 0.918 | 0.839 |
| SVM | 0.922 (0.893 - 0.951) | 0.874 | 0.782 | 0.954 | 0.937 | 0.834 |
| RandomForest | 0.919 (0.887 - 0.950) | 0.879 | 0.771 | 0.974 | 0.963 | 0.830 |
| XGBoost | 0.901 (0.863 - 0.939) | 0.885 | 0.794 | 0.964 | 0.951 | 0.843 |
| LightGBM | 0.911 (0.881 - 0.941) | 0.860 | 0.776 | 0.933 | 0.910 | 0.827 |
| Internal Validation Cohort | | | | | | |
| LR | 0.859 (0.799 - 0.919) | 0.803 | 0.778 | 0.824 | 0.789 | 0.814 |
| SVM | 0.843 (0.779 - 0.908) | 0.803 | 0.667 | 0.918 | 0.873 | 0.765 |
| RandomForest | 0.815 (0.748 - 0.883) | 0.758 | 0.722 | 0.788 | 0.743 | 0.770 |
| XGBoost | 0.793 (0.722 - 0.864) | 0.752 | 0.764 | 0.741 | 0.714 | 0.787 |
| LightGBM | 0.788 (0.718 - 0.858) | 0.745 | 0.556 | 0.906 | 0.833 | 0.706 |
| External Validation Cohort | | | | | | |
| LR | 0.840 (0.759 - 0.922) | 0.806 | 0.789 | 0.814 | 0.698 | 0.877 |
| SVM | 0.828 (0.739 - 0.917) | 0.806 | 0.737 | 0.843 | 0.718 | 0.855 |
| RandomForest | 0.787 (0.687 - 0.888) | 0.759 | 0.763 | 0.757 | 0.630 | 0.855 |
| XGBoost | 0.777 (0.679 - 0.875) | 0.750 | 0.816 | 0.714 | 0.608 | 0.877 |
| LightGBM | 0.793 (0.702 - 0.884) | 0.769 | 0.816 | 0.743 | 0.633 | 0.881 |

AUC, area under the curve; CI, confidence interval; LightGBM, Light Gradient Boosting Machine; LR, Logistic Regression; NPV, negative predictive value; PPV, positive predictive value; RF, Random Forest; SVM, Support Vector Machine; XGBoost, eXtreme Gradient Boosting.

Table S5. Performance of different machine learning algorithms in Clinic model

| Clinic Model | AUC (95% CI) | Accuracy | Sensitivity | Specificity | PPV | NPV |
| --- | --- | --- | --- | --- | --- | --- |
| Training Cohort | | | | | | |
| LR | 0.723 (0.671 - 0.775) | 0.674 | 0.871 | 0.503 | 0.604 | 0.817 |
| SVM | 0.681 (0.626 - 0.736) | 0.655 | 0.824 | 0.508 | 0.593 | 0.767 |
| RandomForest | 0.807 (0.758 - 0.856) | 0.797 | 0.747 | 0.841 | 0.804 | 0.792 |
| XGBoost | 0.789 (0.739 - 0.840) | 0.748 | 0.729 | 0.764 | 0.729 | 0.764 |
| LightGBM | 0.779 (0.729 - 0.830) | 0.751 | 0.635 | 0.851 | 0.788 | 0.728 |
| Internal Validation Cohort | | | | | | |
| LR | 0.709 (0.628 - 0.791) | 0.688 | 0.806 | 0.588 | 0.624 | 0.781 |
| SVM | 0.673 (0.588 - 0.758) | 0.656 | 0.778 | 0.553 | 0.596 | 0.746 |
| RandomForest | 0.731 (0.650 - 0.812) | 0.752 | 0.792 | 0.718 | 0.704 | 0.803 |
| XGBoost | 0.725 (0.641 - 0.809) | 0.694 | 0.458 | 0.894 | 0.786 | 0.661 |
| LightGBM | 0.717 (0.632 - 0.801) | 0.720 | 0.597 | 0.824 | 0.741 | 0.707 |
| External Validation Cohort | | | | | | |
| LR | 0.694 (0.590 - 0.799) | 0.630 | 0.658 | 0.614 | 0.481 | 0.768 |
| SVM | 0.644 (0.533 - 0.755) | 0.657 | 0.421 | 0.786 | 0.516 | 0.714 |
| RandomForest | 0.712 (0.602 - 0.822) | 0.731 | 0.711 | 0.743 | 0.600 | 0.825 |
| XGBoost | 0.666 (0.552 - 0.780) | 0.685 | 0.684 | 0.686 | 0.542 | 0.800 |
| LightGBM | 0.716 (0.613 - 0.819) | 0.704 | 0.711 | 0.700 | 0.562 | 0.817 |

AUC, area under the curve; CI, confidence interval; LightGBM, Light Gradient Boosting Machine; LR, Logistic Regression; NPV, negative predictive value; PPV, positive predictive value; RF, Random Forest; SVM, Support Vector Machine; XGBoost, eXtreme Gradient Boosting.

## 3 Supplementary Figures


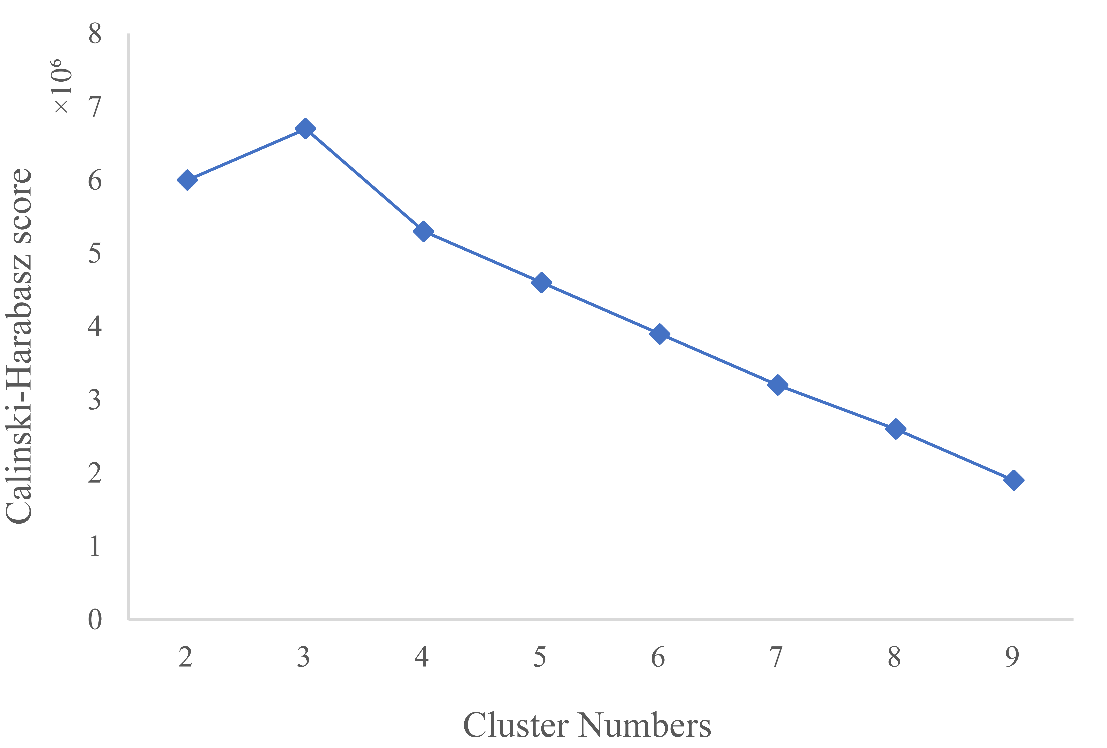


Figure S1. Line plot of Calinski-Harabasz score versus number of clusters


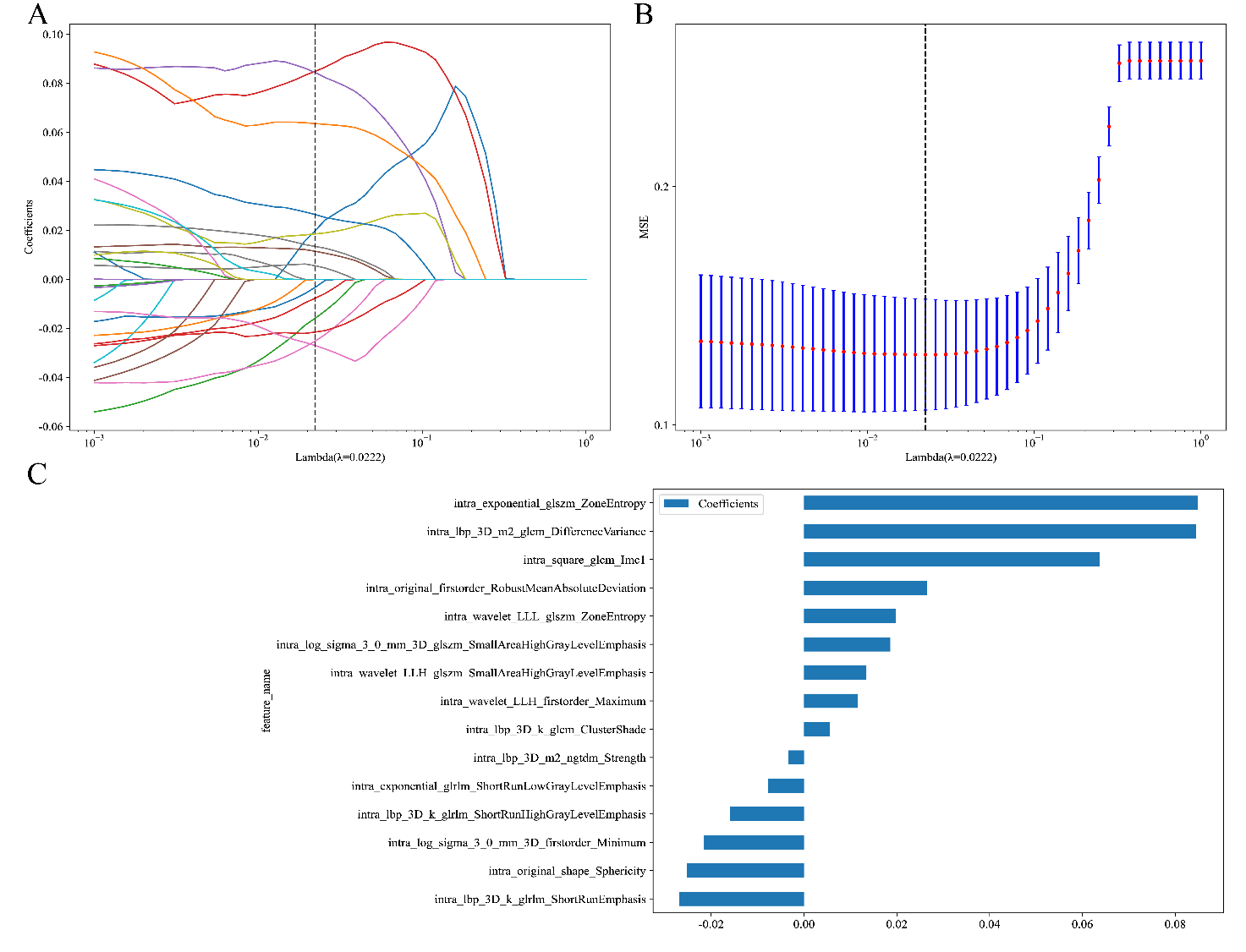


Figure S2. LASSO regression screening of radiomic features in Intra model. (a) LASSO coefficient path plot. This plot illustrates the trajectories of feature coefficients as the regularization parameter (Lambda) varies. As Lambda increases, the coefficients shrink toward zero, identifying key features at the optimal Lambda value (dashed line). (b) LASSO regression MSE curve plot. The dashed line marks the optimal Lambda value where MSE is minimized, determining the final feature subset. (c) LASSO-screened feature coefficient distribution plot. This shows the coefficients of features selected by LASSO regression. MSE, mean squared error.


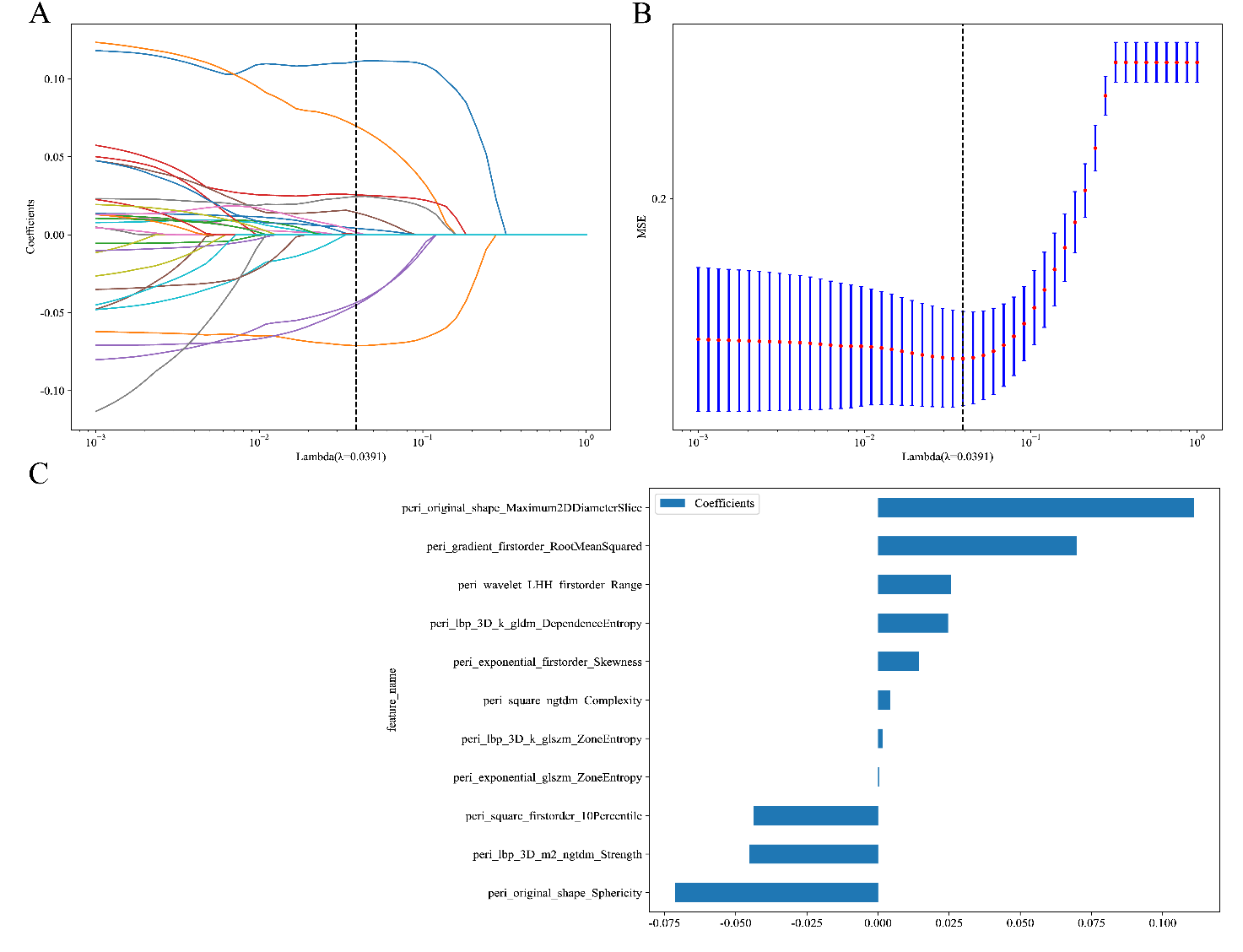


Figure S3. LASSO regression screening of radiomic features in the Peri 1mm model. (a) LASSO coefficient path plot. This plot illustrates the trajectories of feature coefficients as the regularization parameter (Lambda) varies. As Lambda increases, the coefficients shrink toward zero, identifying key features at the optimal Lambda value (dashed line). (b) LASSO regression MSE curve plot. The dashed line marks the optimal Lambda value where MSE is minimized, determining the final feature subset. (c) LASSO-screened feature coefficient distribution plot. This shows the coefficients of features selected by LASSO regression. MSE, mean squared error.


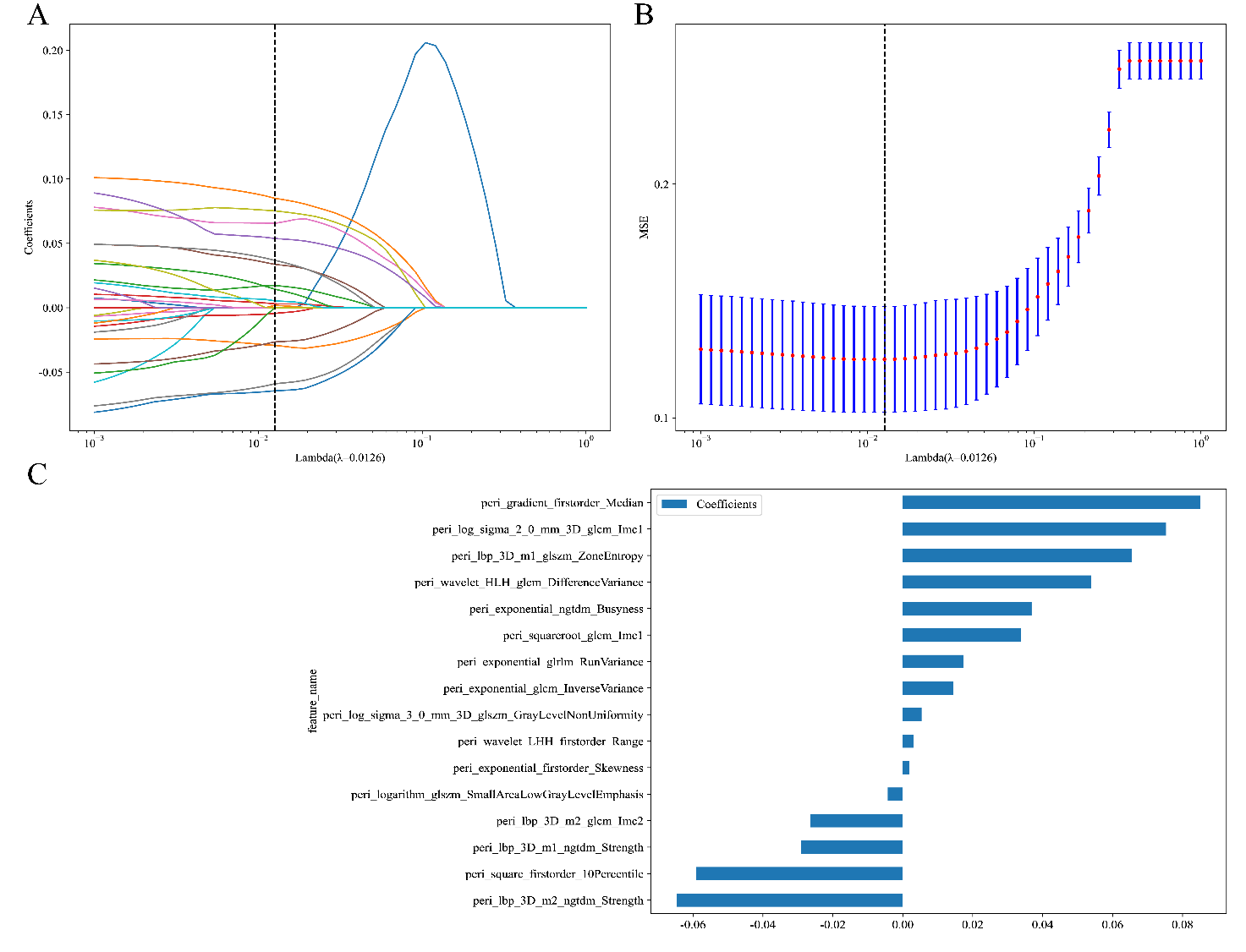


Figure S4. LASSO regression screening of radiomic features in the Peri 2mm model. (a) LASSO coefficient path plot. This plot illustrates the trajectories of feature coefficients as the regularization parameter (Lambda) varies. As Lambda increases, the coefficients shrink toward zero, identifying key features at the optimal Lambda value (dashed line). (b) LASSO regression MSE curve plot. The dashed line marks the optimal Lambda value where MSE is minimized, determining the final feature subset. (c) LASSO-screened feature coefficient distribution plot. This shows the coefficients of features selected by LASSO regression. MSE, mean squared error.


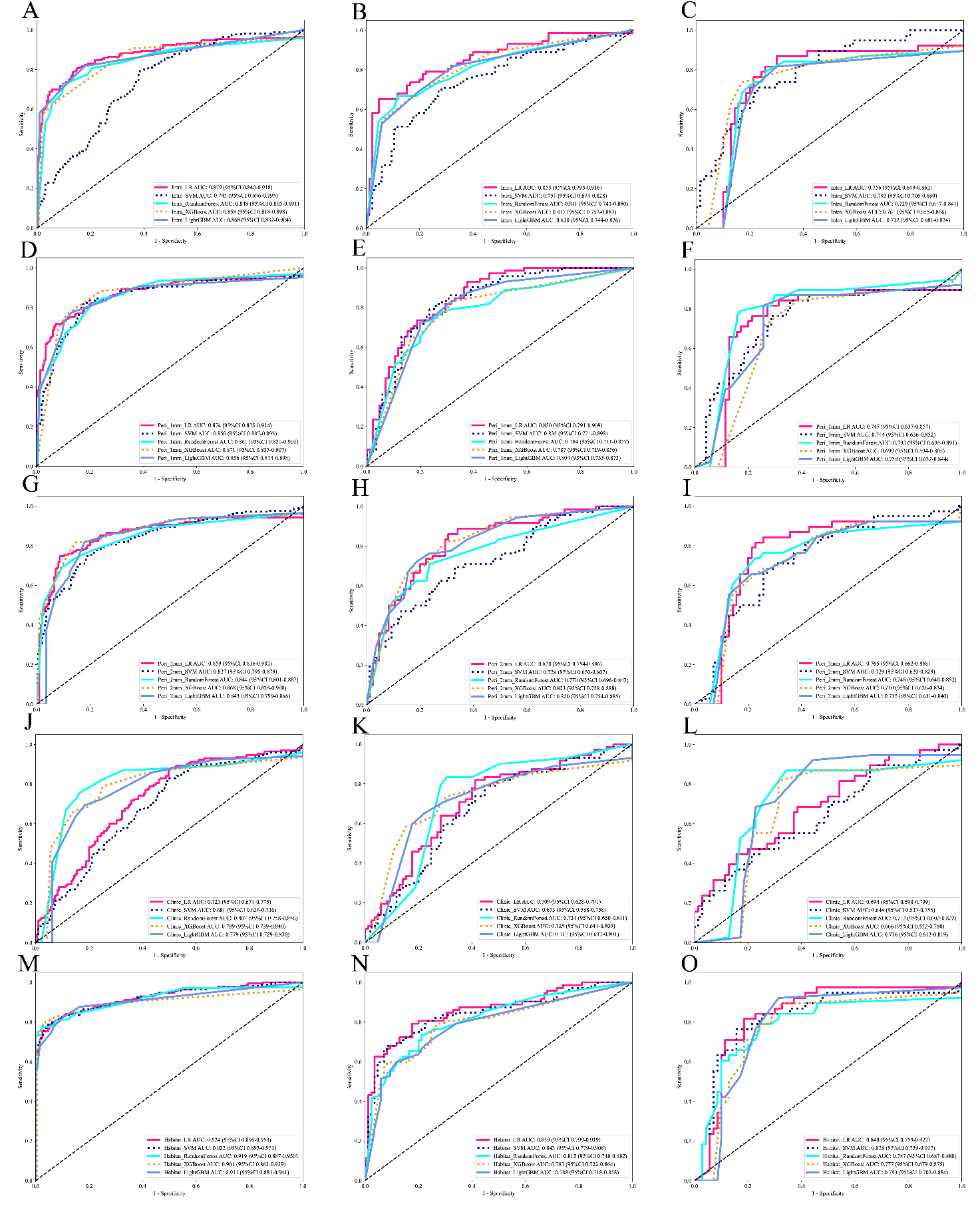


Figure S5. ROC curves of different machine learning algorithms in various models. a-c, ROC curves of different algorithms in Intra model in the training cohort, internal validation cohort, and external validation cohort. d-f, ROC curves of different algorithms in Peri 1mm model in the training cohort, internal validation cohort, and external validation cohort. g-i, ROC curves of different algorithms in Peri 2mm model in the training cohort, internal validation cohort, and external validation cohort. j-l, ROC curves of different algorithms in Clinic model in the training cohort, internal validation cohort, and external validation cohort. m-o, ROC curves of different algorithms in Habitat model in the training cohort, internal validation cohort, and external validation cohort.
